# Feasibility of Flat-Panel CT-Based Perfusion Imaging for Early Diagnosis of Symptomatic Cerebral Vasospasm After Subarachnoid Hemorrhage

**DOI:** 10.7759/cureus.92368

**Published:** 2025-09-15

**Authors:** Kouhei Nii, Naoki Wakuta, Ryuji Matsushita, Koichiro Suzuki, Ritsurou Inoue

**Affiliations:** 1 Department of Neurosurgery, Fukuoka University Chikushi Hospital, Chikushino, JPN

**Keywords:** angiography suite, automated perfusion imaging, diagnosis, flat-panel detector, subarachnoid hemorrhage, symptomatic cerebral vasospasm, vascular imaging

## Abstract

This case study aims to describe the utility of RAPID for ANGIO software (RAPID ANGIO), a flat-panel detector CT-based perfusion and CT angiography-like imaging tool, in the supplementary diagnosis of symptomatic cerebral vasospasm (CVS) following subarachnoid hemorrhage (SAH). Three SAH patients were suspected of having symptomatic CVS, manifested by sudden disturbances of consciousness or focal neurological deficits during the periprocedural period of endovascular treatment (EVT) for ruptured intracranial aneurysms. RAPID ANGIO was used to evaluate vessel narrowing, infarct core, and hypoperfused tissue (defined as time-to-maximum; Tmax >6 seconds), after excluding new hemorrhages using flat-panel CT in the angiosuite. Case 1: A 36-year-old woman developed right hemiparesis and aphasia nine days after SAH. RAPID ANGIO revealed left middle cerebral artery (MCA) narrowing and a substantial penumbra without an infarct core. Angiography confirmed severe vasospasm, and EVT resulted in clinical improvement. Case 2: A 73-year-old woman developed impaired consciousness 11 days after SAH. RAPID ANGIO revealed diffuse right MCA narrowing and mildly delayed perfusion (Tmax >4 seconds). Although EVT was performed, ischemia occurred in the corresponding region. Case 3: A 76-year-old woman experienced transient aphasia 13 days after SAH. RAPID ANGIO revealed no infarct core or penumbra despite possible vessel narrowing. Angiography confirmed the absence of CVS. No further ischemic symptoms occurred. RAPID ANGIO enables rapid, integrated assessment of cerebral perfusion and vascular narrowing in symptomatic CVS. It may serve as a valuable adjunct to guide angiography and EVT decisions in patients with suspected CVS following SAH.

## Introduction

Cerebral vasospasm (CVS) following subarachnoid hemorrhage (SAH) is a major risk factor for delayed cerebral ischemia (DCI), which can result in severe neurological deficits. Therefore, prompt diagnosis and treatment are essential [1]. Angiographic CVS occurs in approximately 30-70% of patients with SAH, with symptomatic CVS developing in 20-30% [1]. When pharmacologic therapy fails to relieve symptomatic CVS, endovascular treatment (EVT), such as intra-arterial administration of vasodilators or percutaneous transluminal angioplasty, is typically performed [2]. Angiography remains the gold standard for determining EVT eligibility; however, because of its invasive nature, it may rarely lead to severe complications [3]. Consequently, adjunctive noninvasive diagnostic modalities for CVS have been explored, including transcranial Doppler ultrasonography (TCD), single-photon emission computed tomography (SPECT), CT angiography (CTA), CT perfusion (CTP), magnetic resonance angiography (MRA), and MR perfusion (MRP) [4-8].

Recently, the utility of automated perfusion imaging software has been demonstrated in determining treatment eligibility for vessel occlusion in acute ischemic stroke [9]. Such software has also been applied in the diagnosis of CVS [7,10]. The ARTIS icono D-Spin (Siemens Healthcare, Erlangen, Germany), an angiographic imaging system, facilitates the acquisition of perfusion and occlusion site data using CTA-like images generated via syngo DynaCT Multiphase and analyzed with RAPID ANGIO (iSchemaView, California, USA) [11]. We report the utility of RAPID ANGIO as an adjunctive diagnostic tool for symptomatic CVS following SAH.

## Case presentation

Between March 2021 and December 2023, EVT was performed for 48 patients with ruptured intracranial aneurysms who presented with SAH. The TCD, conventional CT, and MRI were performed the day after EVT, within one week, and two weeks later. Among them, three were suspected of having symptomatic CVS, manifested by sudden disturbances of consciousness or focal neurological deficits during the periprocedural period. These patients were transferred to the angiosuite, where flat-panel detector CT was used to exclude new intracranial lesions. Subsequently, the location of CVS, infarct core, and hypoperfused tissue (tissue at risk or penumbra) were evaluated using CTA-like images and automated perfusion maps generated with RAPID ANGIO.

RAPID ANGIO was performed as follows: 10 rotational sweeps of the angiographic C-arm system were conducted around the patient during peripheral venous injection of the contrast agent. The first two rotations served as mask runs, and the subsequent eight rotations captured the inflow and outflow of contrast into the cerebral tissue. Approximately five minutes after image acquisition, RAPID ANGIO generated perfusion maps displaying infarct core and penumbra, defined as regions with reduced cerebral blood flow (CBF) and delayed time-to-maximum (Tmax), respectively, compared with normal tissue. The default thresholds for infarct core and penumbra were defined as CBF <45% and Tmax >6 seconds, respectively [11]. Based on clinical symptoms, the location of CVS, and the mismatch between infarct core and penumbra, symptomatic CVS was diagnosed, and additional EVT was performed accordingly.

Case 1

**Figure 1:**
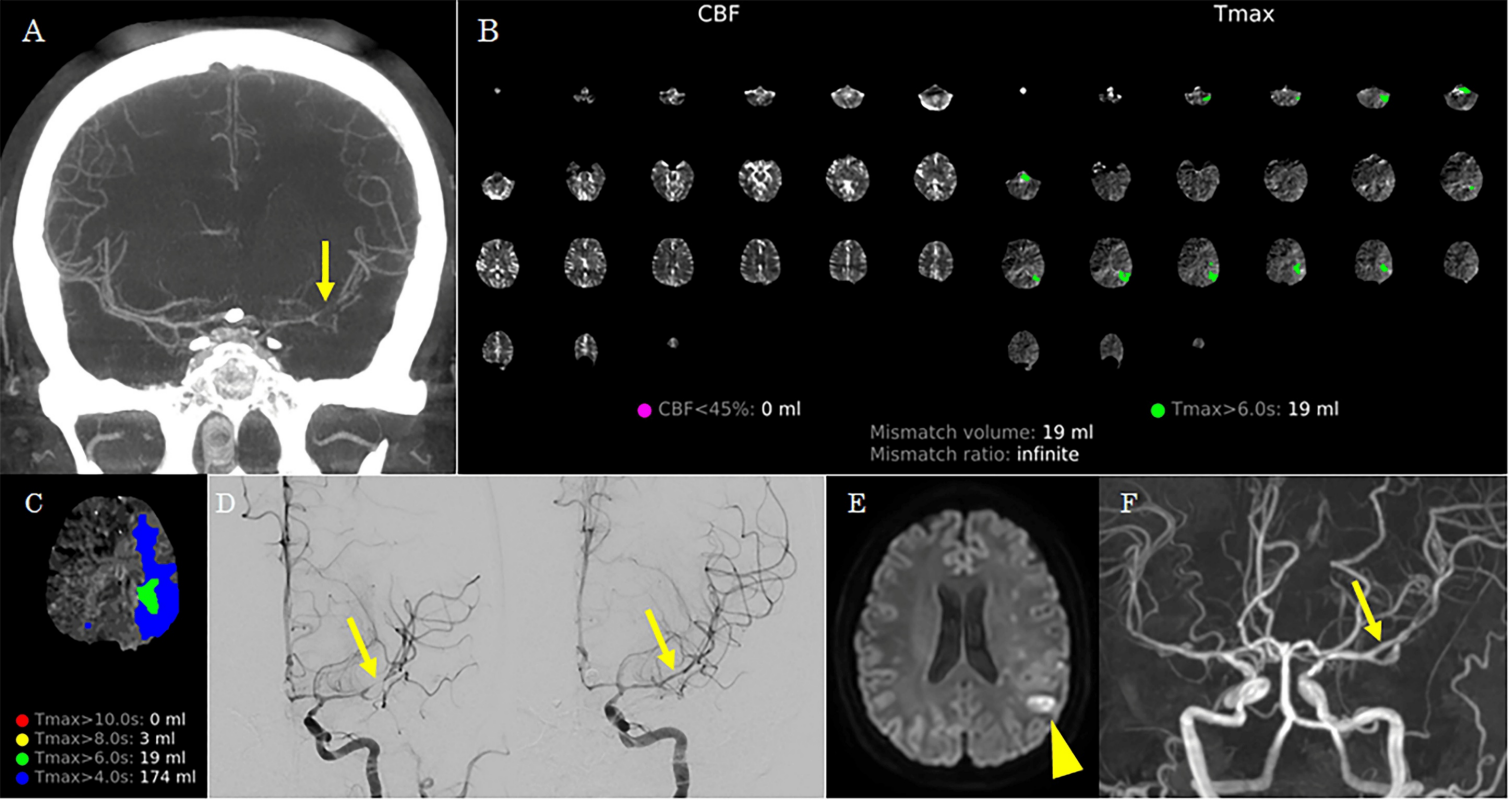
Case 1. (A) CT angiography-like images from the RAPID ANGIO show narrowing of the left middle cerebral artery (arrow). (B) Automated perfusion maps from RAPID ANGIO demonstrate no infarct core (defined as cerebral blood flow <45%) but reveal significant hypoperfused tissue with a time-to-maximum (Tmax) >6 seconds in the left cerebral hemisphere. (C) Detailed Tmax map from the RAPID ANGIO suggests mildly delayed perfusion (Tmax >4 seconds) in the left cerebral hemisphere. (D) Left carotid angiography showing improvement of severe vasospasm in the left middle cerebral artery after intra-arterial administration of fasudil hydrochloride and percutaneous transluminal angioplasty (arrow). (E) Post-procedural diffusion-weighted MRI revealing a small infarction corresponding to the hypoperfused region identified on RAPID ANGIO (arrowhead). (F) Post-procedural MR angiography shows no residual narrowing (arrow).

A 36-year-old woman underwent coil embolization for ruptured basilar artery and right internal carotid artery aneurysms. Post-procedural TCD, CT, and MRI showed negative CVS. The patient was transferred to the angiosuite because of sudden-onset right hemiparesis and motor aphasia nine days after EVT. Flat-panel detector CT revealed no new hemorrhagic lesions. CTA-like images from RAPID ANGIO showed narrowing of the left middle cerebral artery (MCA) (Figure 1A). Automated perfusion maps from RAPID ANGIO demonstrated a penumbra with Tmax >6 seconds in the left cerebral hemisphere, without a significant infarct core (defined as CBF <45%) (Figures 1B, 1C). Follow-up cerebral angiography confirmed vasospasm of the left MCA (Figure 1D). Good reperfusion of the left MCA was achieved through intra-arterial administration of fasudil hydrochloride and percutaneous transluminal angioplasty, resulting in improvement of her right hemiparesis and motor aphasia. Diffusion-weighted MRI following EVT revealed a small infarction corresponding to an area with Tmax >6 seconds on RAPID ANGIO, whereas MRA showed no residual narrowing of the left MCA (Figures 1E, 1F). She was discharged without recurrent CVS 27 days after the final procedure, with a modified Rankin Scale score of 1.

Case 2

**Figure 2:**
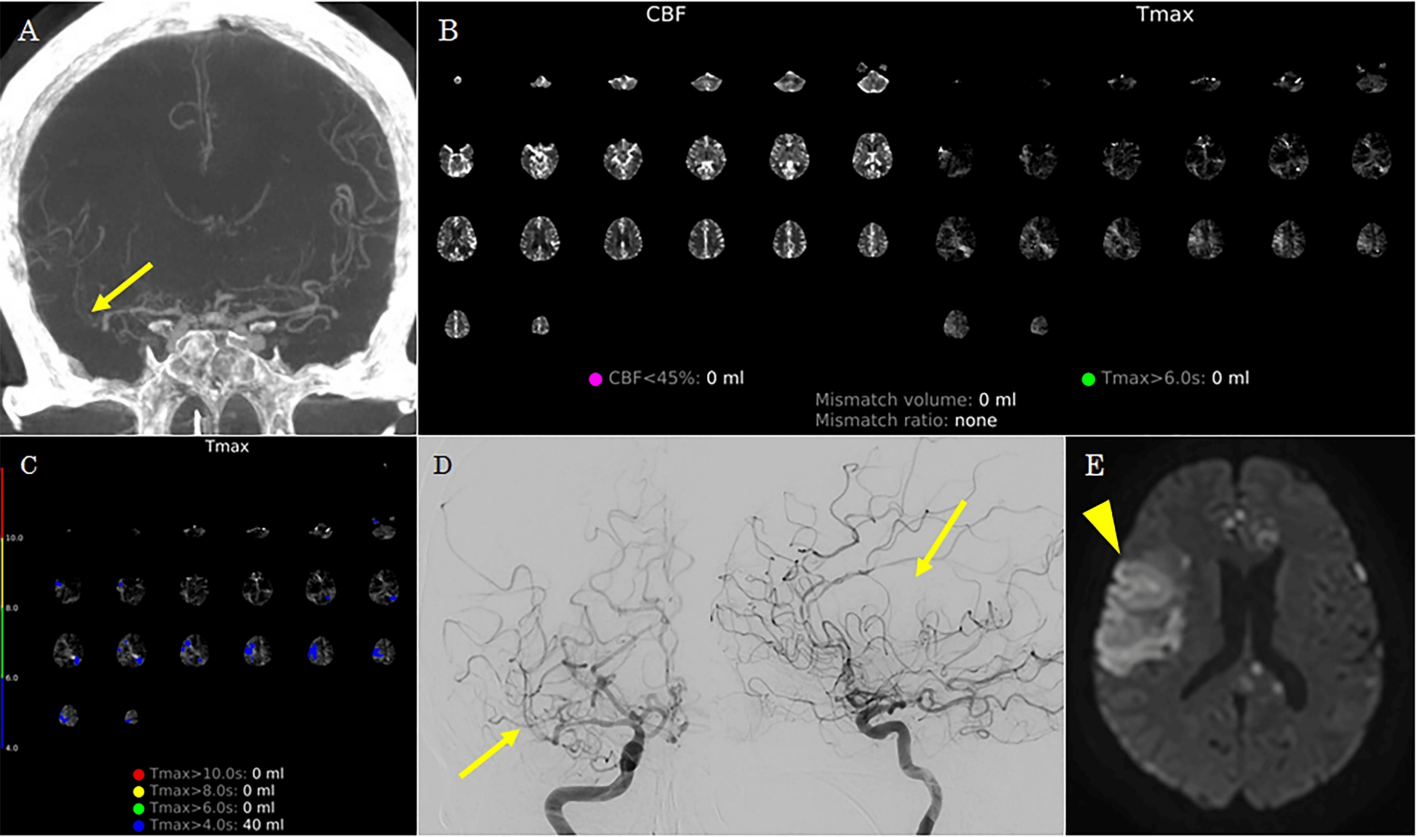
Case 2. (A) CT angiography-like images from RAPID ANGIO showing diffuse narrowing of the right middle cerebral artery (arrow). (B) Automated perfusion maps from RAPID ANGIO show no significant infarct core (cerebral blood flow <45%) or hypoperfused tissue with Tmax >6 seconds. (C) Detailed Tmax maps from the RAPID ANGIO suggest mildly delayed perfusion (Tmax >4 seconds) in the right middle cerebral artery. (D) Right carotid angiography demonstrating diffuse vasospasm of the right middle cerebral artery (arrow), which did not improve following intra-arterial administration of fasudil hydrochloride. (E) Post-procedural diffusion-weighted MRI revealed an infarction in the region of mild hypoperfusion previously suspected in RAPID ANGIO.

A 73-year-old woman underwent coil embolization for a ruptured right anterior cerebral artery aneurysm. Post-procedural examination showed negative CVS. On post-EVT day 11, the patient developed sudden impaired consciousness. Flat-panel detector CT in the angiosuite revealed no new hemorrhagic lesions. CTA-like images demonstrated diffuse narrowing of the right MCA (Figure 2A). Although the perfusion map on RAPID ANGIO did not reveal a distinct infarct core or penumbra (Tmax >6 seconds), mildly delayed perfusion with Tmax >4 seconds was observed in the right MCA territory (Figures 2B, 2C). Follow-up cerebral angiography confirmed diffuse CVS in the right MCA (Figure 2D). Percutaneous transluminal angioplasty was not feasible because of device navigation difficulty; therefore, only intra-arterial fasudil hydrochloride was administered. However, adequate perfusion of the distal right MCA could not be achieved. Diffusion-weighted MRI performed the following day revealed DCI corresponding to the region with Tmax >4 seconds on RAPID ANGIO in the right MCA territory (Figure 2E). The patient exhibited residual moderate impairment of consciousness and left hemiparesis and was transferred to another hospital for recovery rehabilitation three months after the onset of DCI, with a modified Rankin Scale score of 5.

Case 3

**Figure 3:**
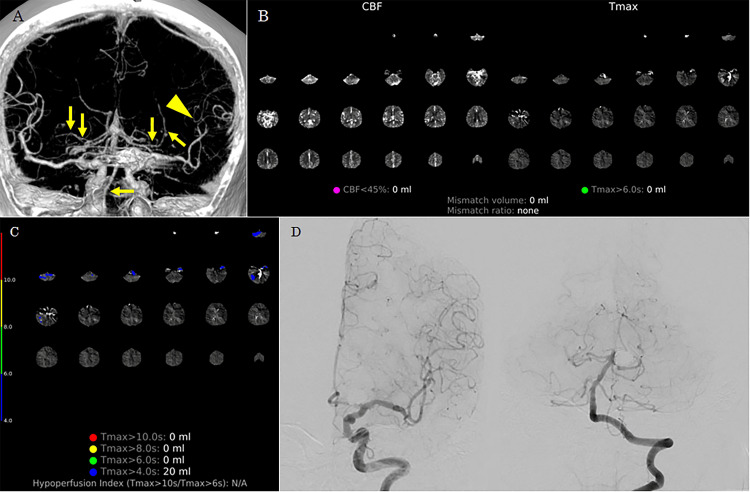
Case 3. (A) CT angiography-like images from RAPID ANGIO suggest possible narrowing of the distal left middle cerebral artery (arrowhead). No vascular narrowing of the posterior circulation was observed (arrows). (B) Automated perfusion maps from RAPID ANGIO show no significant infarct core (cerebral blood flow <45%) or hypoperfused tissue (Tmax >6 seconds). (C) Detailed Tmax maps from RAPID ANGIO suggest mildly delayed perfusion (Tmax >4 seconds) in the posterior circulation. (D) Subsequent left carotid and left vertebral angiography revealed no evidence of cerebral vasospasm.

A 76-year-old woman underwent coil embolization for a ruptured basilar-superior cerebellar artery aneurysm. Post-procedural examination showed negative CVS. She developed aphasia 13 days after EVT. Flat-panel detector CT in the angiosuite showed no new hemorrhagic lesions. Although CTA-like images suggested possible narrowing of the distal left MCA, the perfusion map on RAPID ANGIO did not show a distinct infarct core or penumbra (Tmax >6 seconds) in the left MCA territory (Figures 3A, 3B). Mildly delayed perfusion with Tmax >4 seconds was suspected in the posterior circulation; however, follow-up cerebral angiography did not demonstrate evidence of CVS (Figures 3C, 3D). No symptom recurrence was observed thereafter, and follow-up MRI revealed no DCI. She was transferred to another hospital for recovery rehabilitation 35 days after the onset of SAH, with a modified Rankin Scale score of 2.

## Discussion

Although angiography remains the gold standard for diagnosing CVS, supplementary diagnostic modalities are essential for accurately identifying suspected cases of CVS that warrant the cost and risks of angiography. Rapid diagnosis and treatment are critical to prevent DCI secondary to CVS; thus, diagnostic accuracy, timeliness, and resource efficiency of pre-supplementary diagnostic tools must be considered.

TCD is widely used as a noninvasive screening method to identify patients at risk of CVS. A key advantage of TCD is its ability to be performed daily at the bedside in the intensive care unit. However, it is limited by a narrow field of view and lower sensitivity compared with other modalities [4,7].

SPECT enables regional CBF assessment at the cellular level with minimal risk of image acquisition failure and allows multiplanar visualization. Previous studies have demonstrated the utility of SPECT for early diagnosis and monitoring of CVS [5]. However, semi-quantitative analyses often require normalization to the cerebellum, and the definition of regions of interest may introduce operator-dependent variability [5]. Although SPECT may be more sensitive than angiography for detecting functional changes associated with CVS, it has relatively low specificity for identifying borderline areas associated with DCI [5].

With advances in imaging, both CT and MRI now allow detection of CVS through vascular and perfusion imaging. Vascular imaging modalities such as CTA and MRA enable direct visualization of vasospasm, and numerous studies have demonstrated their utility [3,7,10,12]. However, the reliability of vascular imaging may be compromised owing to reduced interobserver agreement [6].

Perfusion imaging techniques such as CTP and MRP can depict hypoperfused regions caused by CVS [12,13]. CTP is more widely available and frequently reported due to its shorter acquisition time. MRI provides additional capabilities, including arterial spin labeling and the mismatch between perfusion-weighted and diffusion-weighted imaging, to detect penumbral regions [8,14]. Perfusion imaging can also reveal microvascular vasospasm that may not be apparent on vascular imaging alone [15]. Thus, combining vascular and perfusion imaging from the same modality may enhance diagnostic accuracy [6]. Nevertheless, qualitative assessments are subject to interobserver variability, and the optimal perfusion profile for CVS remains undefined.

Tmax, the time to maximum of the deconvolved tissue residue function, has emerged as a robust predictor of hypoperfused and penumbral tissue volumes in acute stroke [16]. Compared with mean transit time, Tmax is less susceptible to patient motion or data undersampling and provides consistent, clearly delineated regions across gray and white matter [16]. Recently, automated analysis software that generates reproducible Tmax maps rapidly and independently of the operator has gained wide adoption in the diagnosis and treatment of acute ischemic stroke [9]. The utility of automated Tmax mapping using CT or MRI has also been demonstrated in CVS after SAH, with improved sensitivity and specificity when combined with vascular imaging [7,10].

To our knowledge, this is the first report describing the use of RAPID ANGIO with flat-panel detector CT in an angiosuite setting for the diagnosis of symptomatic CVS. RAPID ANGIO, which incorporates both perfusion imaging and CTA-like imaging, was considered optimal for CVS assessment. In Case 1, a penumbra with significant perfusion delay (Tmax >6 seconds) was observed along with left MCA narrowing, corresponding to clinical symptoms. Reperfusion therapy resulted in a small asymptomatic DCI. In Case 2, RAPID ANGIO demonstrated mildly delayed perfusion (Tmax >4 seconds) in the right hemisphere, and CTA-like images revealed diffuse narrowing of the right MCA, consistent with clinical findings. Despite intervention, a large DCI developed. In Case 3, mildly delayed perfusion (Tmax >4 seconds) was detected in the posterior circulation, but neither clinical symptoms nor CTA-like abnormalities were present, and subsequent angiography confirmed the absence of CVS or DCI. Similar to CT and MRI, RAPID ANGIO enhances diagnostic accuracy by integrating perfusion and vascular data.

In this study, penumbra was defined as Tmax >6 seconds, based on criteria established in acute ischemic stroke literature [11]. This threshold is also used in other RAPID-based evaluations of CVS using CT and MRI [7,10]. However, the optimal Tmax cutoff for CVS remains debated. Although Tmax >6 seconds indicates clinically significant penumbra warranting reperfusion therapy, some reports suggest that Tmax between 4 and 6 seconds may be more sensitive for early hypoperfusion detection [17]. CTP studies in CVS indicate that Tmax >4 seconds offers higher sensitivity than Tmax >6 seconds, albeit with reduced specificity, suggesting its utility in early DCI prevention [10]. In our cases, vascular narrowing and clinical symptoms could correlate with regions of mildly delayed perfusion (Tmax >4 seconds).

Compared with other diagnostic modalities, RAPID ANGIO offers the advantage of enabling a seamless transition to endovascular treatment. In cases of symptomatic CVS refractory to medical therapy, earlier EVT has been shown to reduce the risk of DCI [18]. Timely EVT is critical, as emphasized in acute ischemic stroke literature, where protocols such as Direct Transfer to Angiosuite (DTAS) have gained interest [19]. The DTAS protocol aims to expedite treatment by bypassing intermediate imaging and transferring patients directly to the angiosuite [19]. In our study, all patients with suspected symptomatic CVS were transferred directly from the ward to the angiosuite, where flat-panel CT was used to exclude intracranial pathology, followed by RAPID ANGIO to assess for CVS. This workflow mirrors the DTAS protocol and may support timely CVS management.

The principal advantage of RAPID ANGIO is its ability to rapidly obtain vascular and perfusion imaging in the angiosuite, similar to CTA + CTP or MRA + MRP, thereby facilitating prompt angiographic diagnosis and EVT for symptomatic CVS after SAH. Although CT- and MRI-based vascular and perfusion imaging have high specificity for diagnosing CVS after SAH, their low sensitivity limits their use for screening [6,10]. RAPID ANGIO also involves mildly invasive procedures, including contrast administration and radiation exposure. Thus, while we caution against its use as a stand-alone screening tool, our findings support the role of RAPID ANGIO as an adjunct for guiding cerebral angiography or EVT in symptomatic cases.

## Conclusions

RAPID ANGIO for symptomatic CVS following SAH may serve as a valuable supplementary diagnostic tool to guide decisions regarding angiography or EVT by combining perfusion and CTA-like images. Furthermore, compared with other diagnostic modalities, RAPID ANGIO offers the additional advantage of facilitating a seamless transition to EVT.

## References

[1] Harrod CG, Bendok BR, Batjer HH (2005). Prediction of cerebral vasospasm in patients presenting with aneurysmal subarachnoid hemorrhage: a review. Neurosurgery.

[2] Elliott JP, Newell DW, Lam DJ (1998). Comparison of balloon angioplasty and papaverine infusion for the treatment of vasospasm following aneurysmal subarachnoid hemorrhage. J Neurosurg.

[3] Francoeur CL, Mayer SA (2016). Management of delayed cerebral ischemia after subarachnoid hemorrhage. Crit Care.

[4] Darsaut TE, Keough MB, Chan AM (2022). Transcranial Doppler velocities and angiographic vasospasm after SAH: a diagnostic accuracy study. AJNR Am J Neuroradiol.

[5] Naderi S, Ozgüven MA, Bayhan H, Gökalp H, Erdoğan A, Egemen N (1994). Evaluation of cerebral vasospasm in patients with subarachnoid hemorrhage using single photon emission computed tomography. Neurosurg Rev.

[6] Heitkamp C, Geest V, Tokareva B (2024). CTA supplemented by CTP increases interrater reliability and endovascular treatment use in patients with aneurysmal SAH. AJNR Am J Neuroradiol.

[7] Sastry RA, Bajaj A, Shaaya EA, Anderson MN, Doberstein C (2022). Utility of automated MRI perfusion (RAPID) with or without MR angiography for detection of angiographic vasospasm after aneurysmal subarachnoid hemorrhage. J Clin Neurosci.

[8] Vatter H, Güresir E, Berkefeld J (2011). Perfusion-diffusion mismatch in MRI to indicate endovascular treatment of cerebral vasospasm after subarachnoid haemorrhage. J Neurol Neurosurg Psychiatry.

[9] Nogueira RG, Jadhav AP, Haussen DC (2018). Thrombectomy 6 to 24 hours after stroke with a mismatch between deficit and infarct. N Engl J Med.

[10] Allen JW, Prater A, Kallas O (2022). Diagnostic performance of computed tomography angiography and computed tomography perfusion tissue time-to-maximum in vasospasm following aneurysmal subarachnoid hemorrhage. J Am Heart Assoc.

[11] Kurmann CC, Kaesmacher J, Cooke DL (2023). Evaluation of time-resolved whole brain flat panel detector perfusion imaging using RAPID ANGIO in patients with acute stroke: comparison with CT perfusion imaging. J Neurointerv Surg.

[12] Greenberg ED, Gold R, Reichman M (2010). Diagnostic accuracy of CT angiography and CT perfusion for cerebral vasospasm: a meta-analysis. AJNR Am J Neuroradiol.

[13] Hertel F, Walter C, Bettag M, Mörsdorf M (2005). Perfusion-weighted magnetic resonance imaging in patients with vasospasm: a useful new tool in the management of patients with subarachnoid hemorrhage. Neurosurgery.

[14] Labriffe M, Ter Minassian A, Pasco-Papon A, N'Guyen S, Aubé C (2015). Feasibility and validity of monitoring subarachnoid hemorrhage by a noninvasive MRI imaging perfusion technique: Pulsed Arterial Spin Labeling (PASL). J Neuroradiol.

[15] Greenberg ED, Gobin YP, Riina H, Johnson CE, Tsiouris AJ, Comunale J, Sanelli PC (2011). Role of CT perfusion imaging in the diagnosis and treatment of vasospasm. Imaging Med.

[16] Calamante F, Christensen S, Desmond PM, Ostergaard L, Davis SM, Connelly A (2010). The physiological significance of the time-to-maximum (Tmax) parameter in perfusion MRI. Stroke.

[17] Olivot JM, Mlynash M, Thijs VN (2009). Optimal Tmax threshold for predicting penumbral tissue in acute stroke. Stroke.

[18] Jabbarli R, Pierscianek D, Rölz R (2019). Endovascular treatment of cerebral vasospasm after subarachnoid hemorrhage: more is more. Neurology.

[19] Requena M, Olivé-Gadea M, Muchada M (2021). Direct to angiography suite without stopping for computed tomography imaging for patients with acute stroke: a randomized clinical trial. JAMA Neurol.

